# Impact of the COVID-19 pandemic on the adaptability and resiliency of school food programs across Canada

**DOI:** 10.3389/fpubh.2023.1296620

**Published:** 2024-01-03

**Authors:** Mavra Ahmed, Alana Richardson, Jessica Riad, Chelsea McPherson, Daniel W. Sellen, Vasanti S. Malik

**Affiliations:** ^1^Joannah and Brian Lawson Centre for Child Nutrition, Temerty Faculty of Medicine, University of Toronto, Toronto, ON, Canada; ^2^Department of Nutritional Sciences, Temerty Faculty of Medicine, University of Toronto, Toronto, ON, Canada; ^3^Department of Nutrition, Harvard T.H. Chan School of Public Health, Boston, MA, United States

**Keywords:** Canada, COVID-19, challenges, children, school food programs, nutrition programs

## Abstract

**Introduction:**

Following the sudden closure of schools due to the pandemic in 2020, many school food program (SFP) operators lost their operating venues and had to innovate to continue distributing meals to children. Our objective was to assess the impact of the COVID-19 pandemic on the delivery, adaptability, and resiliency of school food programs across Canada by conducting a systematic rapid review.

**Materials and methods:**

Systematic literature searches identified newspaper articles and social media sources related to the adaptations and challenges faced by school food programs across Canada in response to the COVID-19 pandemic. Included sources were assessed and thematically categorized according to the dimensions of the Analysis Grid for Environments Linked to Obesity (ANGELO) and Getting To Equity (GTE) frameworks to identify factors impacting the delivery, adaptability, and resiliency of school food programs in Canada.

**Results:**

School food programs in Canada made various efforts to meet existing and new challenges associated with the delivery of these programs to keep feeding school children, particularly those most vulnerable, during the pandemic. Distribution of food kits, prepared meals and gift cards/coupons were successful pathways in ensuring support for food accessibility to students and their families. Increased collaborations between community members and organizations/stakeholders to help maintain food delivery or collectively offer new modes to deliver foods were most frequently cited as key to facilitating school food programming. However, maintenance and sustainability related to operating costs and funding were identified as key challenges to successful school food programming.

**Conclusion:**

Our study highlights the swift and substantial transformation school food programs,, underwent in response to the pandemic, driven by the urgent need to ensure that students still had access to nutritious meals and the importance of policy and resource support to bolster the adaptability and resiliency of these programs. Findings on facilitators and challenges to school food programs during the early months of the COVID-19 pandemic can inform development of guidelines to design a robust national Canadian school food program and help make existing programs more sustainable, adaptable, and resilient.

## 1 Introduction

On March 2020, the COVID-19 outbreak was declared a pandemic by the World Health Organization (WHO) (1), resulting in stresses on economies and food supply chains around the world, and disproportionately impacting the world’s most vulnerable populations, including children. The number of food insecure children nearly doubled due to the COVID-19 pandemic (2). Lockdown measures and school closures during the pandemic are key factors in driving increased food insecurity, by cutting off children’s access to the food sources they once relied on during the school week, especially for children already living in poverty or those with lower socioeconomic status (3).

In Canada, the food retail landscape rapidly evolved during the COVID-19 pandemic through consumer panic buying, increased operating costs of grocery stores (due to new enhanced safety precautions) and potential food shortages as a result of closed manufacturing plants, which led to increases in food prices, particularly for certain core food (i.e., recommended) categories (4). Many Canadians were and are currently faced with reduced work hours or unemployment, lower incomes, and decreased food budgets as a result of the pandemic (5) thereby increasing their risk of food insecurity and poor diet quality. In Canada, as across the world, fractured school operating schedules or long-term school closures mean that many students may no longer have access to meals through voluntary school programs that they may have relied on under normal circumstances to meet their nutritional needs (6). As COVID-19 continues to disrupt the food retail environment and create growing economic uncertainty, children and their families are at increased risk of poor nutrition (6).

School environments that reinforce positive habits and practices are critical in shaping the well-being of children and adolescents, who spend a considerable portion of their day at school. In Canada, children and adolescents reportedly consume about one-third of their daily energy at school, with most of it coming from foods low in nutritional quality (7). While schools could be a channel for nutritious foods through national school food programs, Canada remains one of the few industrialized countries without one (8). Instead, municipal, and provincial/territorial governments and non-governmental organizations support a patchwork of school food programming across Canada (9, 10). Recent evidence suggests that the participation rates of these mixed efforts in school food programming ranges from approximately 5% in Alberta to 83% in Yukon (11). Despite varying participation, school food programs offer a source of quality nutrition for children who may come to school hungry for various reasons (e.g., food insecurity).

School food programs serve nutritious meals and snacks, ensuring consistent access to healthy foods for children. With abrupt school closures due to lockdown measures, many programs faced challenges finding new ways to distribute meals. While news articles captured the adaptation and resilience of school food programs in Canada during the pandemic, there is a lack of systematic evidence synthesis on their emergency adaptations. Therefore, the objective of this study was to conduct a rapid review to systematically explore the impact of the COVID-19 pandemic on the delivery, resiliency, and adaptability of school food programs across Canada, offering insights to strengthen future policies, especially food food-insecure and marginalized communities, particularly during periods of school closures (e.g., during the summer holidays).

## 2 Materials and methods

### 2.1 Information sources and literature search

Recognizing the rapidly evolving nature of the COVID-19 pandemic and its influence on school closures, this study was designed as a systematic rapid review of newspaper articles, social media and grey literature covering information on the delivery, adaptability, and resiliency of school food programs as emergency response feeding strategies across Canada. The research question addressed by this rapid review was: For school-going children and adolescents, how did school food programs adapt during the pandemic in terms of delivery and infrastructure compared with ‘pre-pandemic’ operations, and what lessons were learned from school food programs that were sustained during the pandemic to ensure provision of nutritious meals to students. This study employed a multiple case study approach to guide our data collection, analysis, and interpretation, given the rapidly evolving nature of school food program modalities during the COVID-19 pandemic. Relevant studies were included if there were sufficient access to pertinent data in relation to the research question.

Data extraction was organized using the PICOTS framework as follows: (a) Population – school food programs provided to school-aged children and adolescents; (b) Intervention – various adaptations to school food program modalities; (c) Comparison – “pre-pandemic” school food program modalities; (d) Outcomes – The various items covered under the delivery, adaptability, and resiliency of SFP were adapted from other studies, guided by the RE-AIM framework: Reach and Effectiveness (Delivery) e.g., SFP modalities, food type and variety, method of delivery/food distribution, food procurement, participation/scope. Adoption and Implementation (Adaptability) e.g., infrastructure, changes to content (e.g., menu items, food type/variety/food distribution and procurement) of SFPs. Maintenance and Sustainability (Resiliency) e.g., concerns, challenges and successes encountered, support/beneficiaries, funding and costs and impact; (e) Time – adaptations to school food programs during the COVID-19 pandemic lockdown and (f) Study design/characteristics – case studies, relevant news/webinar articles and inclusion of necessary information with respect to delivery, adaptability and resiliency. This research was guided by two frameworks: the Analysis Grid for Environments Linked to Obesity (ANGELO) (12) and the Getting to Equity (GTE) (13), selected for their complementarity towards capturing the factors impacting the delivery, adaptability, and resiliency of school food programs in Canada.

To identify potentially relevant articles for inclusion, the following electronic databases was searched: ProQuest Canadian NewStream for news articles and Social Search for social media articles. The search was supplemented by grey literature captured through targeted internet searches for news articles, as well as attendance and participation in webinars, that provided additional links to strategies addressing the adaptations of school food programs. A concept map was created to identify relevant keywords (Table 1), and the keywords were further refined with the research team and University librarian in terms of redundancy and ability to capture the relevant articles. The inclusion criteria were any case studies, news articles or webinars that were assessing school food programs (lunch, breakfast, snakes, before/after school) for school-aged children/adolescents and mentioned the delivery, adaptability, or resiliency of these programs during the COVID-19 pandemic in comparison to the regular operating schedule from the time of the public health emergency declaration and school closure announcements in Canada. Studies were excluded if they were looking at day care or early child education center food programs.

**Table 1:** Text used in literature searches.

Literature Search								
School*		Lunch		Program*		COVID-19		Marginalized
Elementary		Breakfast		Policy		COVID*		Insecure
Secondary		Meal*		Environment		Coronavirus*		Vulnerable
High School	**AND**	Nutrient*	**AND**	Arrangement	**AND**		**AND**	At risk
Children*		Snack*		Project				Susceptible
Youth*				Strategy				
Adolescent*				Protocol				
Young Adult*				Supplemental				
Student*								

*Variation each of the text were searched (e.g., school, schools, nutrient, nutrients, snack, snacks, children, child, children’s, kids, youth, adolescents, adolescence, young adults, students etc).

### 2.2 Screening

Articles were eligible if they included keywords related to school food program during the COVID-19 pandemic. Potential sources obtained from literature searches were extracted, organized, and reviewed by two team members (J.R and A.R). The first team member began by screening all potential sources for relevance. Non-relevant sources were excluded (e.g., focusing on school food program adaptations outside Canada), as were any sources that were duplicated. The second team member then reviewed the remaining eligible articles for their inclusion in the study.

### 2.3 Source content analysis and synthesis

The ANGELO framework was designed to assess the environmental factors impacting eating behavior or physical activity and allows for identifying which factors can be readily modified (12). It is designed for communities to identify these factors, however, can be used both at the population (e.g., Canada) and settings/sector level (e.g., schools or fast-food retails). The framework assesses macro and microenvironments, with respect to the physical, economic, political, and sociocultural aspects. Macroenvironments operate at a regional/state level and may include media, food distribution programs, food transport, and food catering services while microenvironments relate to the household/institutional level and may include settings such as the home, school, church, grocery store, and food service outlets (12). In this study, the ANGELO framework was used as a conceptual model to evaluate and assess the balance of the societal and environmental factors impacting adaptability of school food programs during the COVID-19 pandemic (Supplementary Table 1). In reviewing each article, assessors thematically categorized mention of various societal and environmental factors according to the domains of the ANGELO framework.

The Getting to Equity (GTE) framework focuses on equity-oriented obesity prevention action through four types of approaches and was used to assess the gaps in school food programs, specifically for food insecure and marginalized communities during the COVID-19 pandemic (13). The four approaches are: increasing healthy options; reducing deterrents to healthy behaviors; improving social and economic resources; and building community capacity. Increasing healthy options and reducing deterrents focus on potential policy and system interventions that could lead to improved equity, while improving social and economic resources and building on community capacity focus on individual and community resources and capacity developments (13). Specifically, this framework was used to assess the adaptability of school food programs as emergency feeding strategies with a ‘people-oriented perspective’ according to how they affect school-aged children, families, insecure/marginalized communities, other population subgroups and communities. In reviewing each article, assessors thematically categorized the adaptability of school food programs according to the various approaches of the GTE framework (Supplementary Table 2).

Both the ANGELO and GTE frameworks were used to synthesize common themes related to successes and challenges emerging across the adaptability of school food programs in Canada during the COVID-19 pandemic.

### 2.4 Data analysis

In reviewing each article, we thematically categorized mention of various societal and environmental factors according to the domains of the ANGELO and GTE frameworks. The components of each framework were used to synthesize the themes after we thematically analyzed the data using NVivo, guided by the RE-AIM framework. This involved (1) becoming familiar with the literature review/similar studies (14–16), (2) creating initial codes based on these similar studies and using the RE-AIM framework to guide the selection of themes, as relevant to the research question, designed using PICOTS, and (3) Thematically organizing the data.

Data obtained from the search results were extracted and organized into the following categories by each city and province: Reach and Effectiveness (Delivery): SFP modalities (e.g., food kits, gift cards and type of foods), food type and variety, method of delivery/food distribution (e.g., delivery/pickup time), food procurement, participation/scope (e.g., students, families, insecure/marginalized populations). Adoption and Implementation (Adaptability): infrastructure, changes to content/modifications (e.g., menu items, food type/variety/food distribution and procurement) of SFPs (e.g., electronic messages, fund raisers etc.). Maintenance and Sustainability (Resiliency): concerns, challenges and successes encountered, support/beneficiaries, funding and costs and impact. Information was collected and thematically categorized and compiled by the two team members. In case of discrepancy, information was reviewed by a third team member for thematic categorization.

## 3 Results

A total of 166 citations were initially retrieved. Of these, 165 were obtained from ProQuest Canadian Newsstream. The 1 remaining source was obtained from a webinar *Nourishing students: How Ontario SNP’s are adapting in a time of COVID* hosted by Student Nutrition Ontario and sustain Ontario on June 1st 2021.Upon review of the 166 collected citations, 81 duplicated sources and 50 sources deemed to be ineligible for the purposes of this review were excluded from further analysis.

**Figure 1:**
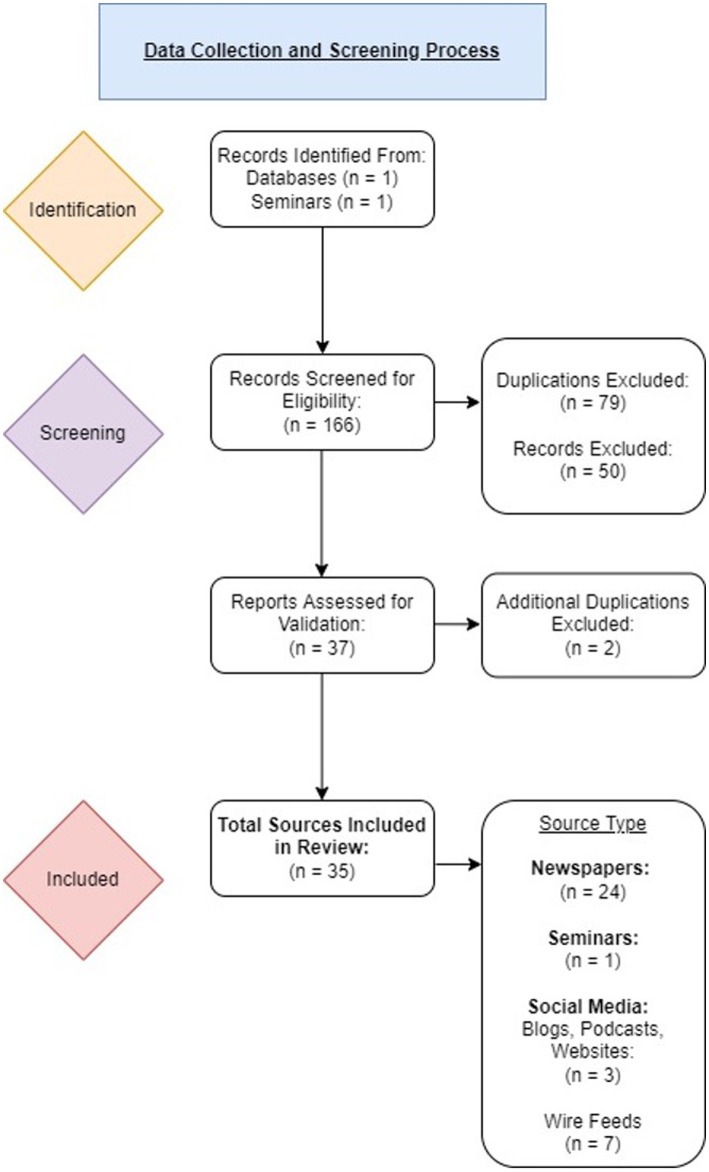
Flowchart of data collection and screening process: A total of 166 citations were retrieved. Thirty five articles from 7 provinces and territories across Canada fulfilled the eligibility criteria and were included in this review. Ten of the included articles were from social media sources.

A total of 35 unique sources were validated and included in this review. The 35 sources consisted of different source types such as newspapers (*n* = 24), seminars (*n* = 1), and social media sources comprised of blogs, podcasts, and websites (*n* = 3) and wire feeds (*n* = 7). Source type was determined using the classifications obtained from ProQuest (excluding the seminar as ProQuest was not used to find this source) (Figure 1).

### 3.1 Captured dimensions of the ANGELO and GTE frameworks

**Figure 2:**
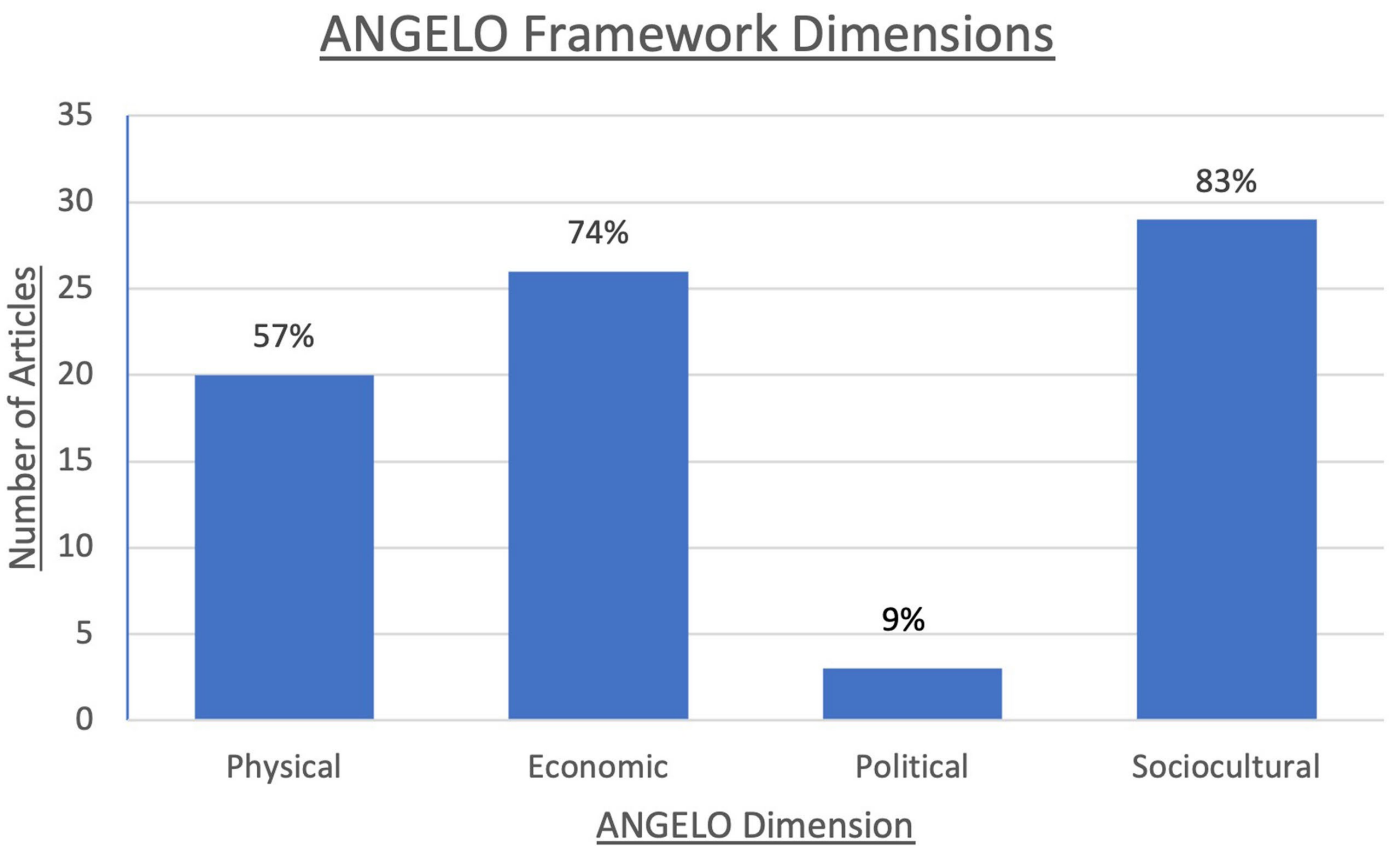
Analysis Grid for Environments Linked to Obesity (ANGELO) framework dimensions captured in included sources: Number of sources (out of total *n* = 35) which captured each respective dimension of the ANGELO framework (12). Examples of physical environment components include food availability, accessibility, and distribution. The economic environment covers aspects such as food costs, price gouging, and available financial resources (e.g., monetary donations, fundraisers) for both SFPs and families in need. The political environment includes governmental interventions such as the creation of new laws, regulations, and funding changes. The sociocultural environment focuses on the attitudes, values, and collaborations of the community and society at large. Abbreviations: ANGELO, Analysis Grid for Environments Linked to Obesity; SFP, School Food Program.

The main dimensions of the ANGELO framework captured by the 35 sources were related to sociocultural (*n* = 29), physical (*n* = 26), or economic (*n* = 20) environments. There was a notable lack of school food program adaptations which incorporated the political environment (n = 3). (Figure 2).

**Figure 3:**
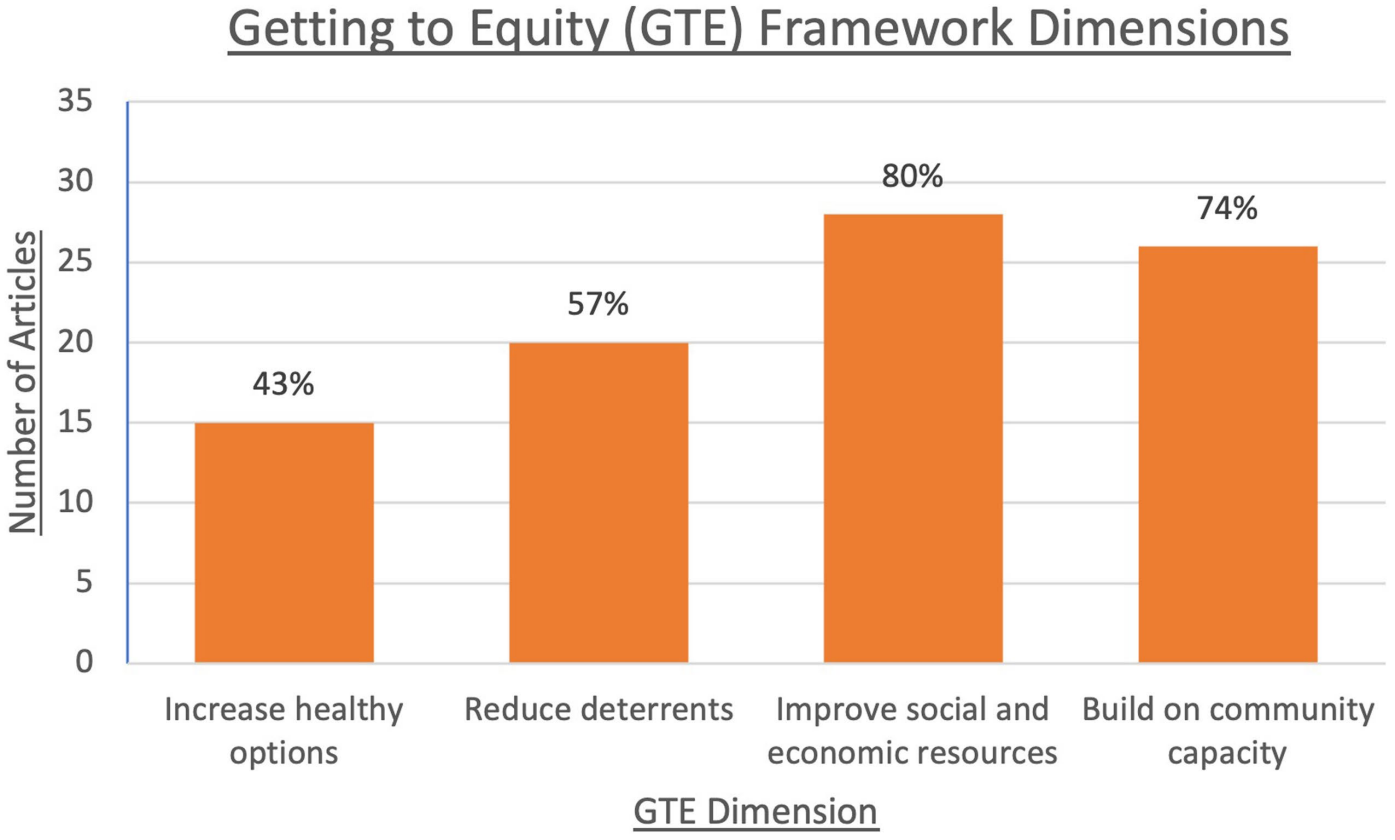
Getting to Equity (GTE) framework dimensions captured in included sources: Number of sources (out of total *n* = 35) which captured each respective dimension of the GTE framework (13). Examples of increasing healthy options includes the distribution of free meal kits and prepared meals which follow existing SFP guidelines, as well as meal kit delivery. Reducing deterrents involves mechanisms such as providing food at accessible locations, following COVID-19 guidelines, and non-discriminatory outreach to all students and families. Improving social and economic resources can occur through the creation of new SFPs during COVID-19, along with increased amounts of funding and donations (e.g., monetary, food, other supplies) to SFPs or directly towards vulnerable students and their families. Building on community capacity can be achieved through collaborations between community members to help those in need, using community resources in beneficial ways, and creating new partnerships to provide greater aid to those in need (e.g., working with larger organizations and companies). Abbreviations: GTE, Getting To Equity, SFP, school food program.

When examined using a basis of the GTE framework, the 35 included sources predominantly focused on improving social and economic resources (*n* = 28) and building community capacity (*n* = 26). Reducing deterrents was also a focus of the included articles (*n* = 20), with the least number of articles focusing on increasing healthy options (*n* = 15) (Figure 3).

### 3.2 Modality of SFP during the pandemic

In terms of modality, novel approaches to the delivery of school food programs included new initiatives or adjustments to old approaches, community initiatives and increased financial support. Programs in several provinces implemented the provision of food kits and gift cards to students, with some even offering home delivery. Community initiatives included setting up shelters or other public locations to provide meals and food kits to students and their families, as well as organizing local events to increase awareness on food insecurity of local families and raise funds. Additionally, with the support of non- governmental organizations (NGOs) and school food program-allocated funds from governments (federal, provincial/territorial), there was greater capacity to maintain emergency food distribution in certain regions (e.g., Nunavut) (Table 2).

**Table 2:** Summary of changes to School Food Programs in response to the COVID-19 pandemic.

Province/Territory *(and Cities)*	Number of Articles Reviewed	Target Demographic(s)	Modalities of COVID-19 Response	Distribution Schedule(s) [for meals/food kits]
New Brunswick *Moncton* (2) *Saint John* (2)	4	Families in need, including families with language barriers	Volunteers partnered with local food banks and other facilities to help continue SFP for 13 weeks in school districts following school closures Creation of emergency food program formed and funded by a collection of local organizations to create and deliver food boxes to food-insecure households	Daily Weekly deliveries
Ontario *Mississauga* *Toronto* (2) *Windsor* *Ottawa* (4) *Clinton* *Thunder Bay*	10	Families in need Fully online learners	Volunteers formed small teams to deliver groceries to families in need during school closures Local restaurants partnered with Ontario School Nutrition Program to become meal kit distribution hub for the areas following school closures Local businesses started making meals for students in need to help provide relief to struggling families following school closures; Local restaurant owner providing meals to children in need in the community Food hamper distribution to aid fully virtual students in need Changing format of in-person fundraising events to virtual in order to continue to raise money for SFPs and local food bank Teachers and volunteers starting new programs to deliver fresh fruits and vegetables to vulnerable students and their families Transition to delivering breakfast meals directly to food agencies to accommodate for school closures and still be able to provide food for families in need Increased funding by the Government of Canada to help initiatives which help combat food insecurity for students and their families Food donations and support by private sectors to partners and distribution of private sector’s products and coupons to most vulnerable families throughout Canada	Daily Weekly
Québec *Montréal*	1	High-risk students	Redistributing funding normally allocated for schools towards students at high-risk for food insecurity/low-income and partners equipped to serve students in need	N/A
Saskatchewan *Maple Creek*	1	School students and staff	Serving fresh meals	Weekly
Alberta *Edmonton* *Edson* *Rycroft* *Sylvan Lake*	4	School students Families struggling with food security	New food distribution program to communities Monetary donations from community to help run SFPs and local food banks during closures Additional government funding to expand SFP into new areas to help more families struggling with food security	Weekly
British Columbia *Vancouver* (3) *Clearwater* *Revelstoke* *Richmond* *Victoria* (4) *New Westminster* *Smithers* *Houston*	13	Families in need of help Students most vulnerable to food insecurity	Community fundraiser to help schools support families in need Creation and funding towards local community project which aims to keep school breakfast programs running Money raised by local groups towards purchasing groceries for families in the community in need of help School district offering free meals to families which normally rely on SFPs for help School staff creating meal kit program for most vulnerable students during closures Principals continuing SFP by personally shopping for food and clothes for students in need and their families Creation of new SFP that provides warm breakfast and meal kit to help struggling families during school closures Local grocery wholesaler donating frozen meat for families in need in absence of SFPs School district setting up distribution hubs at schools to provide weekly meals to vulnerable families Food distribution partnered with new agencies to expand outreach, helping to serve more under-privileged communities during school closures	Twice every week Weekly Every 18 days
Nunavut *Iqaluit*	2	Food-insecure households	Members of Legislative Assembly direct increased amounts of funding towards SFPs to operate during closures Volunteers modified existing breakfast SFP and school food bank to create pre-packaged breakfast kits students could pick up to take home during closures	Daily

SFP, school food program.

### 3.3 Adaptations to school food program coverage

During the pandemic, several school food programs focused on vulnerable students and families, particularly those identified as high-risk of facing food insecurity and/or belonging to marginalized communities (e.g., those with language barriers, minority groups). Most programs adapted to provide meals on a weekly basis, with some in the provinces of New Brunswick and Ontario giving daily options (Table 2).

### 3.4 Types of food kits/prepared meals

For the programs that adapted to provide food kits, the majority of the food kits included some form of fruit, vegetables, dairy and grain products. A few also incorporated granola bars or savory snacks such as crackers. There was none or very limited provision of legumes and pulses, animal protein, sweets and desserts and sugar-sweetened beverages. Only two articles indicated following nutritional guidelines established for the SFP (Table 3).

**Table 3:** Summary of components included in food kits and prepared meals following School Food Programs modifications in response to the COVID-19 pandemic.

Food Kits											
Location	Continued to follow established SFP Nutrition Guidelines?*	Fruits and Vegetables	Legumes and Pulses	Animal Protein (meat/non-dairy)	Dairy	Grains^†^	Savory Snacks^†^	Sweets and Desserts	Sugar-sweetened beverages	Condiments	Other
*Mississauga,* ON				**√**	**√**	**√**					
*Ottawa*, ON		**√**			**√**	**√**	**√**				Granola bars, applesauce
*Thunder Bay*, ON	**√**	**√**			**√**	**√**	**√**				Granola bars
*Toronto*, ON		**√**									
*Windsor*, ON		**√**				**√**					Granola bars
*Smithers*, BC	**√**					**√**					Canned soup, snacks*‡*
*Vancouver*, BC		**√**		**√**		**√**	**√**			**√**	
*Iqaluit*, NV		**√**			**√**	**√**					

Prepared meals§											
Location	Continued to follow established SFP Nutrition Guidelines?*	Examples of Meals Served	Fruits and Vegetables	Legumes and Pulses	Animal Protein (meat/non-dairy)	Dairy	Grains	Savory Snacks†	Sweets and Desserts	Sugar-sweetened beverages	Other
*Moncton*, NB		*Sandwiches* *‡*					**√**				
*Clinton*, ON		*Peanut butter + jelly sandwiches, cereal with milk*				**√**	**√**				Peanut butter, jelly
*Maple Creek*, SK		*Hamburgers, breakfast burritos, chicken, pizza pockets, grilled ham, meatball sandwiches,* *side of fresh vegetables*	**√**		**√**	**√**	**√**				
*Houston*, BC	**√**	*Sandwiches* *‡* *, wraps* *‡* *, side of fruits and vegetables, oatmeal (packaged)*	**√**				**√**				

SFP, school food program.

*Criteria met based on article’s explicit mention of following previously established SFP guidelines and/or consulting higher authority (ex. regional health care provider) about which foods to include in meal kits.

† Examples of grains would be rice or bread. Examples of savory snacks include crackers, tortilla chips and salsa, hummus, and pita, etc.

‡ Relevant articles did not specify the types of snacks, sandwiches, or wraps included/prepared.

§ Meals were either prepared fresh on-site and provided to students or prepared prior, packaged, and distributed.

For the programs that indicated provision of prepared meals, the majority incorporated dairy and grain products as components of meals, while only few indicated the inclusion of animal products other than dairy, fruits, and vegetables (Table 3).

### 3.5 Lessons learned and challenges in implementing modifications to school food programs

One of the factors that was repeatedly mentioned as facilitating the continuation of school food programs during the pandemic was leveraging existing community partnerships and distribution channels. For example, several articles highlighted the collaborative approach that was helpful in swiftly altering the mode of distribution and provision of nutritious meals and snacks, particularly to those families and children in need. This approach was further facilitated by the development of new partnerships with local businesses, which allowed for the purchase of food from local farmers whose distribution chains had been disrupted and local restaurants taking on the responsibility to provide fresh meals to students after losing regular business. Additionally, the flexibility of certain school food programs led to modifications like expansion in food provision range to reach a larger number of children and families, including extra food in meal kits to help support families over the weekend, and transforming restaurants which supplied lunches to one school pre-pandemic into distribution hubs to reach a larger group of families in the area (Table 4).

**Table 4:** Overall summary of positive adaptions made to the School Food Programs in response to the COVID-19 pandemic.

Themes	Ways which positive adaptations emerged across Canada
Collaborations supporting modified SFPs (ex. within community, partnering with organizations)	Donations from community members and local businesses to support preparation of fresh meals/meal kits Volunteers using their own resources and money to continue supporting small group of families who relied on SFPs pre-pandemic Support from local food banks to help keep SFPs supplied during pandemic Formation of new food programs comprised of a collection of organizations to support those struggling with food insecurity Local business owners providing food to children in need by accepting food donations from the community and preparing fresh meals Teachers delivering groceries to families in need, accepting donations from the community to fund grocery purchases Local fire department assisting with meal kit deliveries Private sector donating foods and funding to partners/organizations providing foods
Use of meal kits	Implemented to compensate for lack of fresh meals being served to students during school closures Variety of distribution methods (ex. Delivery to students directly, pick-up at school, pick-up at set distribution points in community) Variable distribution schedules (ex. Bi-weekly, weekly, monthly)
Food types included in meal kits and prepared meals	Meal kits most often contained fresh fruits and vegetables, grain products, dairy products, savory snacks, and products classified as “other” (ex. Granola bars, canned goods) Meal kits lacked sweets and desserts, as well as SSBs Most common prepared meals were sandwiches and wraps (type not usually specified)
Coupon distribution	Private sector working with the Grocery Foundation of Canada to provide private sector’s product coupons to families in need across the country
Price gouging and food affordability	Meal kits and fresh meals provided free of charge by school/organizations to students in need and their families
Flexibility of SFPs	Extra food provided in meal kits to help support families over the weekend (and beyond) Local restaurant which normally supplied lunches to one school becoming a distribution hub for meal kits to help larger group of families in the area Changing normal school meal distribution format to meal kit delivery to still provide food to families in need despite school closures Expansion of SFP into new areas to reach larger number of students and families in need Some SFPs accounted for the food preferences and dietary needs of students
Financial resources used to support continuation of SFPs during the pandemic	Donations from organizations supporting delivery of groceries to most vulnerable students and their families Community fundraisers normally held in-person moved to an online format in order to raise money for local food initiatives Members of legislative assembly (MLAs) allocating increased amounts of spending towards SFPs using emergency budget Individual members of community starting fundraisers towards helping local SFPs (ex. woman climbing mountains to raise money) Fundraisers through grocery stores used to help local schools purchase appliances, cutlery, food gift cards, etc. for cafeteria and SFP Grocery gift cards provided to vulnerable families who relied on SFPs pre-COVID 19 Government of Canada allocating immediate funding towards supporting SFPs
COVID-19 guideline adherence	Volunteer staff wearing proper PPE (ex. Face mask, gloves) when preparing and distributing prepared meals/meal kits Meal kits prepared with precautions for easy disposal and minimal contact (ex. wrappings, paper bag) Physical distancing procedures followed (ex. one person picks up meal kit from distribution location)
Novel ideas	Focusing on decentralized practices to recover economic and socialized institution practices (ex. using experiential education settings such as a farm to offer alternative teaching methods to small groups of students and provide fresh food to students and the community)

SFP, school food program.

A shared barrier among the school food programs were the increased expenses involved with the procurement of food and meals, and additional operating costs related to adhering to COVID-19 protocols. Challenges faced when adapting school food programs also included a heavy reliance on community donations and volunteers, as well as the use of electronic devices and social media to reach families and children in need, although not all families had internet access. Several school food programs continued providing healthy meals during the pandemic without the provision of additional resources. In addition, they faced issues such as limited food supplies and interruptions to food transportation (Table 5).

**Table 5:** Challenges faced by modified School Food Programs during the COVID-19 pandemic.

Themes	Ways which challenges emerged across Canada
Use of meal kits	Certain methods of meal-kit delivery (ex. by local fire department) were only available during certain days/weeks – uncertainty faced when trying to co-ordinate other modes of delivery
Food types included in meal kits and prepared meals	• Meal kits commonly lacked animal protein, legumes and pulses Prepared meals were usually low in fruits and vegetables and/or animal protein (or did not specify if these foods were included) Very few meal kits/prepared meals followed established SFP guidelines on foods to include and serve
Price gouging and food affordability	Increased difficulties faced when trying to access food due to increased prices, lack of donations from usual community donors
Flexibility of SFPs	Changes to SFPs (ex. changing provider) led to loss of food choice, increased negative attitudes of students towards the SFP, and loss of personal relationships between students and SFP staff (ex. changing to externally prepared and catered format) Some modified SFPs relied solely on electronic communication and social media announcements which served as a barrier to students and families without internet access
Financial resources used to support continuation of SFPs during the pandemic	Expenses of modified SFPs hard to maintain, especially when run by small team of volunteers – rely on community donations to continue and have strict budget School district having to cover costs to maintain SFP instead of relying on donations Funding uncertainties make it unclear as to how long the modified SFPs will last since it is not sustainable to be run by volunteers alone

SFP, school food program.

## 4 Discussion

**Figure 4:**
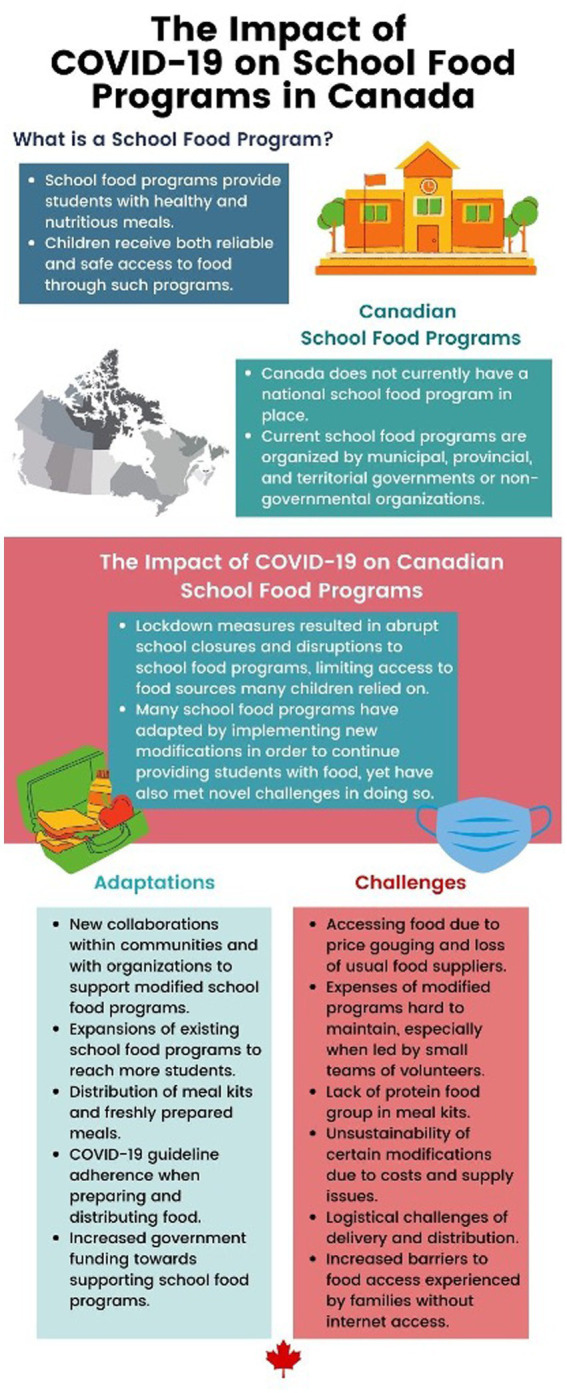
Summary on Canadian school food programs and examples of different adaptations and challenges: Summary of the main adaptations and challenges faced by school food programs across Canada in response to the COVID-19 pandemic as evident through this systematic rapid review.

The findings from this study illustrate how voluntary school food programs across Canada modified and adapted feeding strategies during school closures and the facilitators and challenges they faced in delivering essential provisions to school-aged children, particularly those who were the most vulnerable, which resulted from the COVID-19 pandemic (Figure 4). There was an increased emphasis on community engagement, identified as a key component in facilitating provision of foods to children. However, despite the strengthened efforts among community members, a lack of or limited funds allocated for emergency feeding made delivery of foods challenging, as related to maintenance and sustainability.

As shown from the results of this study, these programs devised and implemented new modalities, which included a mixture of meal provision strategies such as meal pick up at a central location or direct delivery of food kits/prepared meals or gift cards to use in grocery stores. Results from other countries such as the US, Europe or Latin America assessing emergency feeding response indicated similar adaptations (e.g., food kits/ food vouchers) to feeding modalities (14, 17, 18). The results from this study on types of modalities are in alignment with another Canadian study examining the breadth of school food programming; indicating the provision of meals (e.g., breakfast), food boxes and gift cards to sustain school food programming during school closures (16). Direct delivery of food kits/prepared meals or specified pick-up locations ensured student food accessibility, particularly for those who may have limitations accessing these locations via public/personal transport methods. These adaptations to program accessibility can also benefit students with food provision during the ‘summer’ months or when schools are not in session, particularly for students receiving free or reduced-priced meals during the school year. Recent studies have incorporated spatial analyses to assess school meal accessibility related to geographic opportunity, finding that meal distribution sites were often located in larger high poverty areas and areas with a higher proportion of visible minorities (19–21).

Increased outreach (e.g., through local events) and expansion of existing programs (to those outside the community via internet/social media campaigns) also helped improve student food access, as captured by the ANGELO framework of sociocultural factors and GTE framework of building community capacity. Considering the associated risks to student health and well-being due to disruptions in food access, particularly students from vulnerable groups who may lack other nutritious options, future research in Canada can benefit from examining proximity to food access points in relation to factors such as race/ethnicity and socioeconomic status.

The development and effectiveness of these new modalities depended on resource availability and logistical navigation for acquiring and distributing foods with an emphasis on securing funds for sustainability. Support from NGOs, private organizations and/or governmental support was identified as contributing to the maintenance of these emergency food distributions, particularly in regions that are difficult to access (e.g., Nunavut). The modifications in school food programs reflected an increased emphasis on improving social and economic resources as captured by GTE and physical/economic environments aspects as captured by ANGELO. The emphasis on adequate and sustained resources has also been echoed by school-level program volunteers to ensure program feasibility and fidelity (22). These resources not only indicate a need for more staff and enhanced funding but also for resources to train and support staff (e.g., educational workshops to handle food waste or food for large numbers of children) (22).

Considering the patchwork of programming and multi-modality strategies as adaptations in various regions across Canada, it is unclear whether one method of modality is preferred, feasible or functional over another and how they might be associated with contextual factors such as stigma. A 2017 study investigating using food vouchers or free daily lunch (to reduce food insecurity) found the daily lunch method was preferred by many families, with less stigma associated with this method in comparison to food vouchers (23). However, use of a particular modality is likely community specific as factors identified in this study, such as accessibility and reach, are likely to play a role in their success.

Similar to the response by other countries (14, 17, 18), many organizations considered alternative innovative strategies to continue meal delivery. For example, distribution sites such as local community centers, places of worship and other public institutions were used to maximize access. Home delivery was another innovative approach with a high uptake in neighborhoods and for hard-to-reach areas. This suggests the potential to expand the school food programs beyond the traditional modes of delivery, for school-aged children in remote/hard-to-access areas and for when schools are not in session. Similar benefits have been shown in other studies indicating that relaxing restrictions or expanding locations of free meal sites can increase child access to meals during expected and unexpected disruptions to programs and continue to ensure access to nutritious food (24). In many areas, school meals were extended beyond school-aged children to unhoused individuals/families, those relying on food banks, food insecure families and other vulnerable population groups.

Collectively, this suggests that cross-sectoral collaborations to serve children and families can help strengthen community engagement and local partnerships to resourcefully deploy feeding strategies across various population groups, reaching the most at-risk populations. The results from this study are similar to a case study showing that student nutrition programs rich in partnerships enables the staff to pivot and respond efficiently and quickly to the lockdown restrictions (25). Additional studies have also demonstrated the importance of community/multisectoral partnerships in the provision of local, healthy, and traditional foods for schools (26, 27). Importantly, such partnerships have potential for long-term sustainability and maintenance of school food programs.

Despite logistical challenges, some of the programs identified aimed to provide foods as recommended by 2007 Canada’s Food Guide, e.g., including some form of vegetables, fruits, dairy and grain products. However, the challenges of a decreased food supply, price gouging, and changes to normal program delivery impacted the provision of fresh foods and led to increased provision of processed shelf-stable foods that are typically high in discretionary nutrients. Difficulties related to following recommended school food program guidelines were likely related to challenges obtaining fresh food and desire to limit food perishing as much as possible. This is also made evident through the lack of articles which reflected the GTE framework dimension of increasing healthy options. Additionally, food accessibility for children with allergies and/or certain dietary restrictions was more difficult due to the decreased variety of foods available in meal kits and limited options of prepared meals due to indicated challenges. Other countries also reported similar challenges in the provision of healthy foods (14, 17, 18). For example, in UK, media reports showed that many food parcels/kits were inadequate and did not meet school food standards with parents criticizing a lack of fruits and vegetables and inadequate portion sizes (28).

Several studies have indicated that meals offered through school food programs can make substantial contributions in helping children meet their dietary recommendations (29–31), with research indicating consumption of school meals positively relating to child intake of key food groups (e.g., school food participants were more likely than nonparticipants to consume milk, fruit, vegetables and less likely to consume desserts and snack items) (32, 33). Canada’s Food Guide stresses the importance of consuming products with healthy fats and limiting highly processed foods (34), thus continued efforts are needed to ensure school meals offered in Canadian schools meet nutrition standards. In addition, beyond food accessibility and availability, children may also benefit from nutrition education programs to facilitate adoption of better food choices and encourage healthy food-related behaviors (35).

In addition to challenges associated with the quality of foods, other barriers that were commonly mentioned included difficulties in reaching vulnerable families, financial constraints/available funds and limited government fund allocation and involvement in emergency feeding. Students without internet access may have faced limitations in participating in programs relying on online communication. Limited funding raised uncertainties about the sustainability of volunteer-run food programs, impacting the number of families assisted weekly. There was a notable lack of political intervention (e.g., few instances of increased government funding) as captured by the ANGELO framework (the ‘political’ environment factor was only captured by 3 sources). This highlights a critical need for increased government support of school food programs and feeding strategies during emergencies/school closures; this increased support would also help decrease uncertainties/unknowns associated with school food program reliance on donations and volunteers and would also help ensure that students/families, particularly those in need, have reliable access to healthy food for defined or prolonged periods, mitigating stress associated with food and nutrition insecurity (15).

Canada is the only Organization for Economic Co-operation and Development (OECD) country without a school food program. In the UK, for example, which has a national school food program,, schools were required to adapt their approach to school meals due to COVID-19 to ensure support for eligible children (15, 17) and in the US, which also has a national school food program, United States Department of Agriculture waivers were authorized for provision of school meals (18, 24). Despite not having a national school feeding strategy, various approaches were adapted across Canada to reach school-aged children, particularly those in greatest need. These findings highlight key adaptations to school food programs that played a vital role in responding to student and family food needs and underscore the challenges of feeding children during emergency situations or when schools are closed for prolonged periods.

Unhealthy dietary behaviors are a major preventable risk factor for obesity. Canadian children have poor diet quality (36, 37), consuming diets that are high in sodium, sugar and saturated fat ((36)). Considering that schools are an important setting to address childhood obesity and support healthy eating behaviors (30, 38), implementation of school-based nutrition interventions to create healthy school food environments can benefit student health and well-being. School food programs in Canada continue to be provided by a patchwork of programming and could benefit from a comprehensive and standardized approach that addresses nutritional standards, accessibility and equitable distribution to ensure all students have access to healthy and balanced meals (39).

Considering the national and global momentum on the importance of school food programs for meeting nutritional needs and ensuring access to healthy foods for children (40, 41), the results from this study can be contextualized to highlight lessons learned for future considerations for school food program implementation in Canada and during future emergencies and/or during school closures, as follows: (1) establishment of national school food guidelines to guide the nutritional quality of foods, (2) allocation of funds/resources dedicated to provision of school feeding strategies, and (3) capacity building for strengthening community engagement and local partnerships.

### 4.1 Establishment of national school food guidelines

Recommendations to reduce intake of foods with nutrients-of-concern (sodium, sugar and saturated fat) and processed foods are recommended by health organizations worldwide, given the increase in overweight, obesity and diet-related non-communicable diseases (42). The current study indicated, that although there were attempts made at provision of foods from various food groups including fruits and vegetables, limited articles mentioned following nutritional guidelines when determining the types of food provided to children (also given the constraints with resourcing, price increase of foods, and distribution/delivery etc.). Nutritional guidelines are critical to avoid providing less healthful, nutrient-poor foods in emergency programs. Previous studies have indicated prevalence of high-calorie, low nutritional quality foods in such assistance efforts (14, 43). Additionally, self-stable ultra-processed foods, easily pre-packaged for individual consumption, may be provided without assessing their nutritional adequacy in emergency responses (14, 43). Considering the limited literature on the nutritional quality of foods provided during emergencies in high-income countries, there is a critical need for national nutritional guidelines for school feeding programs that also address provision during, school closures, and future emergencies.

### 4.2 Allocation of funds/resources dedicated to provision of feeding strategies

Limited funds and resources hindered schools from sustaining food provision during COVID-19 related school closures, despite innovative strategies, community engagement and donations. The patchwork of funding coming from various sources and uncertainty surrounding the continuation of funds led to cessation of many feeding adaptations shortly after being initiated, emphasizing the need for a sustainable funding structure for school food programs (44). This challenge extends beyond Canada, as budget constraints were observed globally and noted as a significant barrier to the operability of feeding strategies during the pandemic in other countries (14, 17), highlighting the political aspects of the ANGELO domain with inconsistent funding allocation.

### 4.3 Capacity building for strengthening community engagement and local partnerships

Adapting school feeding strategies successfully involved community engagement and forming local partnerships for food resourcing and access. Collaborations, such as volunteers working with local food banks and/or community organizations, played a vital role in the adaptability, sustainability, and resiliency of school feeding strategies. Future design of school feeding strategies should prioritize partnerships between local community organizations and government support to enhance community capacity. Previous studies from the US and Latin American emphasize the importance of local partnerships as key to the success for the adaptability of school feeding strategies (14, 18). Strengthening community engagement may also contribute to the success of complementary community food security responses to reach the most vulnerable.

### 4.4 Strengths and limitations

This study is limited by using case selection and non-traditional literature sources for information on adaptability of school feeding strategies during the COVID-19 pandemic school closures, particularly, as many organizations diverted to use of social media to raise awareness and information about feeding strategies. Data from news articles, gathered through rapid assessment, may not capture specific challenges faced by organizations and/or communities. This study may also be limited in identifying the full breadth of feeding strategies reaching marginalized communities. Nonetheless, the findings highlight challenges and facilitators, informing future considerations. Comparisons with school food programs in other countries such as the US or UK is limited due to Canada lacking a national school feeding strategy. As such, certain aspects of this research, e.g., whether the grocery store gift cards were used for food only is beyond the scope of this study.

This study is the first of its kind in Canada to capture the adaptability and resiliency of various school food programs by province and city during an emergency and provides an overview of the challenges and facilitators faced in adapting school food programs. Furthermore, considering the rapid assessment approach, this study applied validated frameworks (ANGELO AND GTE) that assessed food environments, to give a snapshot of where future considerations could focus for emergency and/or out-of-school feeding strategies.

## 5 Conclusion

The findings highlight key recommendations that can help inform government and civil society to more readily face the challenges associated with school feeding during similar emergency periods and/or during sustained periods of school closure (i.e., during summertime). Drawing from the lessons learned, the findings underscore the importance of establishing national nutrition guidelines to meet nutritional needs of children, allocation of immediate/emergency funds to sustain the provision of school food programs and supporting strengthening of community efforts to increase the accessibility and affordability of healthy foods within communities.

## Data availability statement

The raw data supporting the conclusions of this article will be made available by the authors, without undue reservation.

## Author contributions

MA: Conceptualization, Formal analysis, Investigation, Methodology, Supervision, Writing – original draft, Writing – review & editing. AR: Methodology, Visualization, Writing – original draft, Writing – review & editing. JR: Formal analysis, Methodology, Visualization, Writing – original draft, Writing – review & editing. CM: Conceptualization, Methodology, Project administration, Supervision, Writing – review & editing. DS: Conceptualization, Funding acquisition, Investigation, Project administration, Supervision, Writing – review & editing. VM: Investigation, Methodology, Project administration, Supervision, Validation, Writing – review & editing.
