# Supplementary material for: Impact of the COVID-19 pandemic on the adaptability and resiliency of school food programs across Canada
Source: Front Public Health. 2024 Jan 3;11:1296620. doi: 10.3389/fpubh.2023.1296620 (PMC10791823; doi:10.3389/fpubh.2023.1296620)
Supplement: Supplementary file 1 [file Table_1.DOCX]

Supplementary Material

**Supplementary Table 1:** Adaptation of ANGELO framework as a conceptual model to assess balance of societal and environmental factors (adapted from Swinburn et al., (12)).

| **Domain** | **Scale** |
| --- | --- |
| **Physical** | What is available in terms of physical structures (e.g., building, amenities, facilities)? Includes built environments, landscapes and internal/enclosed spaces  e.g., closures of school, distribution/delivery adaptations, via other institutions |
| **Economic** | What are the monetary cost factors/ influences/ consequences? Includes price incentives or disincentives.  e.g., food insecurity/financial resources of families within communities/ financial support, funding towards emergency feeding, gift cards |
| **Political** | What are the rules/legal guidelines, statutory provisions/ political message? Includes acceptable practices/behaviours.  e.g., recommendations/guidelines in place for emergency feeding or for nutritional quality of foods, allocation of resources/funds |
| **Socio-cultural** | What are the attitudes, beliefs, perceptions, and values? Includes cultural/community norms  e.g., community engagement, initiatives, and awareness |

**Supplementary Table 2:** Adaptation of GTE framework to assess factors affecting targeted populations and communities (adapted from Kumanyika., (13)).

| **Categories** | **Scale** |
| --- | --- |
| **Increase Healthy Options** | Potential policy and system interventions that could lead to improved equity e.g., nutritional guidelines, types of foods, procurement of foods |
| **Reduce Deterrents to Health Behaviours** | Potential policy and system interventions that could lead to improved equity e.g., community involvement, awareness and fund-raising campaigns, delivery to people/communities in needs, new modality options |
| **Improve Social and Economic Resources** | Individual and community resources and capacity developments  e.g., expansion of operations, building/developing extended partnerships |
| **Build Community Capacity** | Individual and community resources and capacity developments  e.g., local partnerships, community engagement, initiatives, and awareness |
